# Frequency and Echocardiographic Correlates of Right Ventricular Dysfunction in Patients With Old Myocardial Infarction and Reduced Ejection Fraction

**DOI:** 10.7759/cureus.114476

**Published:** 2026-08-13

**Authors:** Kaoser Alam, Mohammad Monzurul Alam Bhuiyan, Farzana Rahman, Menhazul Islam, Chowdhury Rehnuma Tabassum, Kazi Azajul Ferdoush, Md. Hasan Iqbal Majumder, Sushil Kumar Ray, Chaudhury Meshkat Ahmed

**Affiliations:** 1 Cardiology, Bangladesh Medical University, Dhaka, BGD; 2 Laboratory Medicine, Bangladesh Medical University, Dhaka, BGD; 3 Haematology, Bangladesh Medical University, Dhaka, BGD; 4 Radiology, Bangladesh Medical University, Dhaka, BGD; 5 Paediatric Neurology, Bangladesh Medical University, Dhaka, BGD

**Keywords:** echocardiography, heart failure with reduced ejection fraction, myocardial infarction, right ventricular dysfunction, right ventricular strain, speckle-tracking echocardiography

## Abstract

Background: Old myocardial infarction (MI) with reduced left ventricular ejection fraction (LVEF) is a major cause of cardiovascular morbidity and mortality, particularly in low- and middle-income countries. While left ventricular dysfunction has been extensively studied, right ventricular (RV) involvement remains underappreciated despite evidence of its prognostic relevance in other cohorts. This study aimed to determine the frequency of RV dysfunction and its echocardiographic correlates in patients with old MI and reduced LVEF.

Methods: This was a single-centre, hospital-based, observational cross-sectional study conducted over 12 months (April 2021-March 2022) in the Department of Cardiology, Bangladesh Medical University (formerly Bangabandhu Sheikh Mujib Medical University), Dhaka, Bangladesh, following ethical approval. Adults aged >30 years with old MI documented by objective criteria, occurring more than three months before enrolment, and LVEF <40% were recruited by consecutive sampling from inpatient and outpatient services. The primary outcome was the frequency of RV dysfunction, defined a priori for each index as tricuspid annular plane systolic excursion (TAPSE) <17 mm, tricuspid annular systolic velocity (S′) <10 cm/s, RV fractional area change (FAC) <35%, myocardial performance index (MPI) ≥0.55, isovolumic relaxation time (IVRT) >30 ms, isovolumic acceleration (IVA) <1.1 m/s², RV global longitudinal strain (RVGLS) less negative than −17%, and RV free wall strain (RVFWS) less negative than −20%. The secondary outcome was the association between elevated pulmonary artery systolic pressure (PASP >35 mmHg) and each abnormal RV index. Binary logistic regression was performed with each RV index modelled separately as the dependent variable.

Results: Of 195 patients screened, 85 were analysed (mean age 59.9 ± 8.1 years; 81.2% male; mean LVEF 32.1 ± 4.1%). Reduced TAPSE and S′ were each present in 62.4%, reduced FAC in 56.5%, elevated MPI in 55.3%, prolonged IVRT in 57.6%, and reduced IVA in 31.8%. Impaired RVGLS and RVFWS were observed in 76.5% and 71.8%, respectively. PASP >35 mmHg was detected in 38.8% and was associated with abnormal S′ (OR 6.39; 95% CI 1.11-36.73; p = 0.038) and prolonged IVRT (OR 8.79; 95% CI 1.72-44.93; p = 0.009). Regional RV wall motion abnormality was not associated with any RV functional index.

Conclusion: RV dysfunction is highly prevalent in patients with old MI and reduced LVEF. Abnormality was most frequent for strain-based indices, particularly RVGLS; however, no comparative discrimination or reclassification analysis was performed, so relative diagnostic performance against conventional indices cannot be inferred from these data. Comprehensive RV assessment should be considered in this high-risk population.

## Introduction

Heart failure remains a major global health challenge, contributing substantially to morbidity, mortality, and healthcare expenditure. Ischaemic cardiomyopathy following prior myocardial infarction (MI) is a leading cause of heart failure with reduced ejection fraction (HFrEF) worldwide, including in low- and middle-income countries such as Bangladesh. While clinical evaluation has traditionally emphasised left ventricular (LV) systolic function, accumulating evidence indicates that right ventricular (RV) dysfunction is also closely linked to disease progression and outcomes in ischaemic heart disease and HFrEF [1,2].

Despite its clinical significance, RV dysfunction is frequently under-recognised in routine practice. The right ventricle differs from the left ventricle in structure and physiology and is particularly susceptible to changes in afterload and pulmonary pressures. Chronic LV dysfunction may impair RV performance through mechanisms including ventricular interdependence, pulmonary hypertension due to left heart disease, and sustained neurohormonal activation [3,4]. Importantly, RV dysfunction independently predicts adverse outcomes, including higher mortality, recurrent hospitalisations, and reduced functional capacity in chronic heart failure [5,6].

Echocardiography is the cornerstone for non-invasive assessment of RV function. Conventional indices such as tricuspid annular plane systolic excursion (TAPSE), fractional area change (FAC), and tissue Doppler-derived systolic velocity (S′) are widely used but may be less sensitive for early or subclinical RV impairment [7]. The 2025 American Society of Echocardiography (ASE) guideline for right-heart assessment, which supersedes the 2010 recommendations, restructures RV evaluation around three domains (structure, function, and haemodynamics) and replaces single binary cut-offs with graded severity categories for RV basal diameter, wall thickness, right atrial size, TAPSE, FAC, and S′ [8]. It further emphasises that echocardiography assigns a probability of pulmonary hypertension, based principally on peak tricuspid regurgitation velocity together with supportive right-heart findings, rather than establishing the diagnosis, and gives RV free wall longitudinal strain and three-dimensional RV assessment a more prominent role [8]. Speckle-tracking echocardiography enables quantification of RV longitudinal strain, particularly RV free wall strain, using standardised deformation imaging definitions and consensus guidance [9,10]. RV strain has shown greater sensitivity and prognostic utility compared with conventional measures in multiple cohorts [11,12]. However, much of the evidence is derived from Western populations, and data from South Asian settings remain limited.

In Bangladesh, ischaemic heart disease is an increasing public health burden, yet information on the prevalence, echocardiographic profile, and correlates of RV dysfunction among patients with old MI and HFrEF is scarce. This study evaluated the frequency of RV dysfunction using both conventional and advanced echocardiographic techniques and examined its clinical and echocardiographic correlates among patients with old MI and reduced LVEF attending a tertiary cardiac care centre.

## Materials and methods

Study design and setting

This was a single-centre, hospital-based, observational cross-sectional study conducted in the Department of Cardiology, Bangladesh Medical University (formerly Bangabandhu Sheikh Mujib Medical University), Shahbagh, Dhaka, Bangladesh. The study period was 12 months, from April 2021 to March 2022.

Study population and definition of old myocardial infarction

Adult male and female patients aged >30 years attending inpatient or outpatient cardiology services were screened. Old (prior) myocardial infarction was defined according to the Fourth Universal Definition of Myocardial Infarction [13], requiring at least one of the following: (i) a documented previous hospital admission for acute myocardial infarction supported by a rise and/or fall of cardiac troponin accompanied by ischaemic symptoms or ischaemic electrocardiographic changes; (ii) pathological Q waves on the resting 12-lead electrocardiogram in a recognised coronary territory, with or without symptoms, in the absence of a non-ischaemic cause; (iii) echocardiographic evidence of regional loss of viable myocardium, with thinned, akinetic, or dyskinetic segments in a coronary distribution; or (iv) documented obstructive coronary artery disease on prior coronary angiography, with or without previous revascularisation. To ensure the infarction was established rather than recent, only patients whose index infarction had occurred more than three months before enrolment were eligible; this interval was verified from hospital records, discharge summaries, or angiographic reports. Eligible patients also required echocardiographic evidence of reduced LVEF (<40%) and provided written informed consent.

Exclusion criteria

Patients were excluded if they presented with acute myocardial infarction or acute coronary syndrome; had an index infarction less than three months before screening; or had moderate-to-severe primary (organic) valvular heart disease, a prosthetic heart valve, a permanent pacemaker, congenital heart disease, severe pulmonary disease, active infection, or a chronic debilitating illness. Secondary (functional) mitral regurgitation, being an expected consequence of left ventricular remodelling in this population, was not an exclusion criterion. Pregnant women were excluded, as were those who declined to participate or were unable or unwilling to undergo the required investigations. Patients in whom image quality was inadequate for speckle-tracking analysis were excluded after enrolment.

Sampling and participant flow

**Figure 1:**
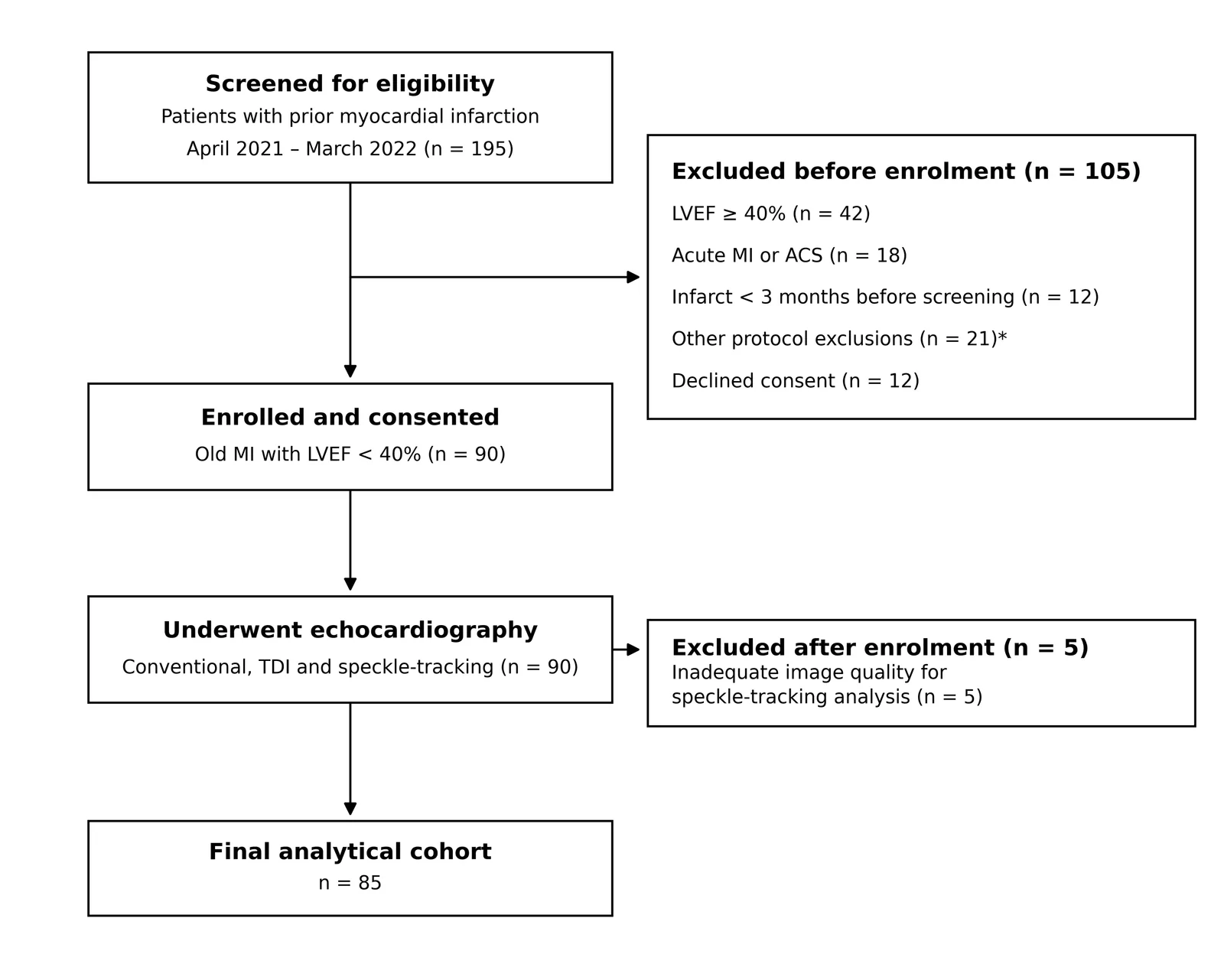
Participant flow through screening, enrolment, and analysis Flow of participants from screening to the final analytical cohort. Of 195 patients with a documented history of myocardial infarction screened between April 2021 and March 2022, 105 were excluded before enrolment and 90 were enrolled and underwent echocardiography. Five were excluded after enrolment because of inadequate image quality for speckle-tracking analysis, yielding a final analytical cohort of 85 patients. Other protocol exclusions comprised moderate-to-severe valvular disease or prosthetic valve (n = 7), permanent pacemaker (n = 4), congenital heart disease (n = 3), severe pulmonary disease, active infection or chronic debilitating illness (n = 5), and pregnancy (n = 2). ACS: acute coronary syndrome; LVEF: left ventricular ejection fraction; MI: myocardial infarction; TDI: tissue Doppler imaging.

Participants were enrolled by consecutive sampling. A total of 195 patients with a documented history of myocardial infarction were screened during the study period. Of these, 105 were excluded before enrolment: 42 had LVEF ≥40%, 18 presented with acute myocardial infarction or acute coronary syndrome, 12 had an infarct less than three months before screening, 21 met other protocol exclusions (moderate-to-severe primary valvular disease or prosthetic valve, n = 7; permanent pacemaker, n = 4; congenital heart disease, n = 3; severe pulmonary disease, active infection, or chronic debilitating illness, n = 5; pregnancy, n = 2), and 12 declined consent. Ninety patients were enrolled and underwent echocardiography; five were subsequently excluded because of inadequate image quality for speckle-tracking analysis. Eighty-five patients constituted the final analytical cohort (Figure 1).

Sample size

In the absence of a reliable prior prevalence estimate for right ventricular dysfunction among patients with old myocardial infarction and reduced ejection fraction in this population, the sample size was estimated using the following formula for a single proportion, applying the maximum-variance assumption:

$n = \frac{Z^{2}p(1&minus;p)}{d^{2}}$

With Z = 1.96 for a 95% confidence level, p = 0.50, and a permissible absolute error d = 0.11, the minimum requirement was 80 participants. Ninety patients were enrolled, and 85 completed all study procedures.

Clinical assessment and coronary angiography

After enrolment, participants underwent history taking and physical examination. Clinical data included demographics (age, sex, and body mass index), cardiovascular risk factors (smoking or tobacco use, hypertension, diabetes mellitus, dyslipidaemia, and family history of coronary artery disease), and heart failure symptoms (dyspnoea graded by New York Heart Association (NYHA) class, peripheral oedema, and fatigue). The type of index infarction was classified from prior admission records and the resting electrocardiogram as ST-elevation or non-ST-elevation myocardial infarction and by anatomical territory, and the interval since the index event was recorded.

Laboratory investigations included complete blood count, fasting and postprandial blood glucose, glycated haemoglobin, renal function tests, serum electrolytes, calcium, magnesium, uric acid, thyroid function tests (thyroid-stimulating hormone, free T3, and free T4), and N-terminal pro-B-type natriuretic peptide (NT-proBNP). Estimated glomerular filtration rate (eGFR) was calculated using the Chronic Kidney Disease Epidemiology Collaboration equation, and anaemia was defined by World Health Organization criteria (haemoglobin <13 g/dL in men and <12 g/dL in women). Medication use at enrolment was recorded by class, comprising renin-angiotensin system inhibitors (angiotensin-converting enzyme inhibitor, angiotensin receptor blocker, or angiotensin receptor-neprilysin inhibitor), beta-blockers, mineralocorticoid receptor antagonists, sodium-glucose cotransporter-2 inhibitors, loop diuretics, statins, and antiplatelet agents.

Left heart catheterisation was not a protocol requirement. Coronary angiography had been performed previously as part of routine clinical care in 58 of 85 patients (68.2%), of whom 34 had undergone percutaneous coronary intervention and 12 had undergone coronary artery bypass grafting; in the remainder, prior infarction was established by the electrocardiographic, biomarker, and imaging criteria described above.

Echocardiography

Comprehensive transthoracic echocardiography was performed using a GE Vivid E95 system (GE Healthcare, Horten, Norway) with standard two-dimensional, M-mode, and Doppler techniques. Left ventricular assessment included LVEF, LV end-diastolic and end-systolic volumes, regional wall motion abnormalities, and chamber quantification in line with established recommendations [14]. Left ventricular diastolic function was evaluated using guideline-based Doppler and tissue Doppler parameters, including transmitral inflow indices (E/A) and E/e′ [15]. Mitral regurgitation severity was graded by integrated multiparametric assessment.

Right ventricular function was assessed using TAPSE, tricuspid annular peak systolic velocity (S′), RV FAC, MPI, IVRT, IVA, estimation of PASP, and inferior vena cava diameter and collapsibility, following professional society recommendations for right-heart assessment [7,8]. Cut-off values applied were those current at the time of data collection: TAPSE <17 mm, S′ <10 cm/s, FAC <35%, MPI ≥0.55, IVRT >30 ms, and IVA <1.1 m/s² [7,14]. PASP was estimated non-invasively from the peak tricuspid regurgitation jet velocity using the modified Bernoulli equation, with the addition of right atrial pressure derived from inferior vena cava diameter and respiratory collapsibility, and categorised into predefined bands (<35, 35-50, 51-70, >70 mmHg), with PASP >35 mmHg considered elevated [7].

RVGLS and RVFWS were acquired by two-dimensional speckle-tracking echocardiography and reported using consensus-based deformation imaging definitions and standardisation guidance [9,10]. Strain was considered abnormal at RVGLS less negative than −17% and RVFWS less negative than −20%, in keeping with published reference values [16]. Additional parameters included ventricular interdependence, assessed from the pattern of interventricular septal flattening in systole, diastole, or both; intraventricular dyssynchrony, defined by a septal-to-posterior wall motion delay ≥130 ms; interventricular dyssynchrony, defined by an interventricular mechanical delay >40 ms [17]; left ventricular geometry, characterised by the eccentricity index in diastole and at end-systole [14]; and regional right ventricular wall motion abnormality, assessed qualitatively in standard apical and subcostal views [7,14].

Speckle-tracking analysis was performed offline using EchoPAC version 204 (GE Healthcare, Horten, Norway), with images acquired at frame rates of 50-80 frames per second. All echocardiographic indices used are standard, guideline-defined measurements described by international professional societies [7-10,14], and no separate licensing is required to apply these definitions in clinical research.

Reproducibility and blinding

All echocardiographic examinations were performed and analysed by a single experienced echocardiographer using a standardised acquisition and measurement protocol. The operator was blinded to laboratory results, including NT-proBNP, and to clinical characteristics other than those required to establish eligibility, namely left ventricular ejection fraction and the presence of prior infarction; complete blinding was not feasible given the enrolment criteria. Formal intra- and inter-observer reproducibility analysis was not undertaken.

Statistical analysis

Data were analysed using IBM SPSS Statistics for Windows, Version 23 (Released 2015; IBM Corp., Armonk, New York). Normally distributed continuous variables were expressed as mean ± standard deviation and skewed variables as median with interquartile range (IQR); categorical variables were expressed as frequencies and percentages. Categorical comparisons were performed using the chi-square test. The primary outcome was the frequency of right ventricular dysfunction across the pre-specified indices; the secondary outcome was the association between elevated PASP (>35 mmHg) and each abnormal right ventricular index. Binary logistic regression was performed with each right ventricular index modelled separately as the dependent variable. Models contained a single predictor and were therefore unadjusted; residual confounding cannot be excluded. The significance of each predictor was assessed using the Wald test, with results reported as the Wald χ² statistic (df = 1), odds ratio (OR), 95% confidence interval (CI), and two-sided p-value. A p-value <0.05 was considered statistically significant.

## Results

Baseline characteristics

The mean age of the cohort was 59.9 ± 8.1 years (range 32-75), and 69 patients (81.2%) were male. The index infarction had occurred a median of 14.0 months (IQR 6.0-32.0) before enrolment and was an ST-elevation myocardial infarction in 56 patients (65.9%); the inferior territory was most frequently involved (48.2%). Coronary angiography had been performed previously in 58 patients (68.2%), with percutaneous coronary intervention in 34 (40.0%) and coronary artery bypass grafting in 12 (14.1%), leaving 39 patients (45.9%) without prior revascularisation. Tobacco use (70.6%), diabetes mellitus (69.4%), dyslipidaemia (68.2%), and hypertension (51.8%) were the most prevalent risk factors, and renal impairment was common, with eGFR below 60 mL/min/1.73 m² in 62.4%. Most patients were symptomatic, with NYHA class II or III dyspnoea in 81.2% and reduced exercise tolerance in 95.3%. Uptake of guideline-directed medical therapy was high for renin-angiotensin system inhibitors (80.0%) and beta-blockers (85.9%), lower for mineralocorticoid receptor antagonists (54.1%), and lowest for sodium-glucose cotransporter-2 inhibitors (44.7%). Mean LVEF was 32.1 ± 4.1%, with a mean end-diastolic volume of 162.4 ± 34.2 mL; median NT-proBNP was 1840 pg/mL (IQR 850-3420), and moderate functional mitral regurgitation was present in 21 patients (24.7%) (Table 1).

**Table 1:** Baseline demographic, clinical, and echocardiographic characteristics of the study population (n = 85) *Mitral regurgitation was secondary (functional) in all cases; patients with moderate-to-severe primary (organic) valvular heart disease were excluded. ACEi: angiotensin-converting enzyme inhibitor; ARB: angiotensin receptor blocker; ARNI: angiotensin receptor–neprilysin inhibitor; eGFR: estimated glomerular filtration rate; HbA1c: glycated haemoglobin; IQR: interquartile range; LV: left ventricular; LVEF: left ventricular ejection fraction; NT-proBNP: N-terminal pro-B-type natriuretic peptide; NYHA: New York Heart Association; RAS: renin–angiotensin system; SD: standard deviation; SGLT2: sodium–glucose cotransporter-2; WHO: World Health Organization.

Characteristic	Value
Demographics	
Age, years, mean ± SD	59.9 ± 8.1
Age range, years	32–75
Male, n (%)	69 (81.2)
Female, n (%)	16 (18.8)
Body mass index, kg/m², mean ± SD	23.4 ± 2.6
Normal body mass index, n (%)	73 (85.9)
Myocardial infarction history	
Interval since index infarction, months, median (IQR)	14.0 (6.0–32.0)
ST-elevation myocardial infarction, n (%)	56 (65.9)
Non-ST-elevation myocardial infarction, n (%)	29 (34.1)
Infarct territory (electrocardiographic)	
Inferior, n (%)	41 (48.2)
Anteroseptal, n (%)	22 (25.9)
Anterior, n (%)	16 (18.8)
Anterior and inferior, n (%)	6 (7.1)
Revascularisation	
Prior coronary angiography, n (%)	58 (68.2)
Percutaneous coronary intervention, n (%)	34 (40.0)
Coronary artery bypass grafting, n (%)	12 (14.1)
No revascularisation, n (%)	39 (45.9)
Cardiovascular risk factors	
Ever-smoker or tobacco user, n (%)	60 (70.6)
Diabetes mellitus, n (%)	59 (69.4)
Dyslipidaemia, n (%)	58 (68.2)
Hypertension, n (%)	44 (51.8)
eGFR <60 mL/min/1.73 m², n (%)	53 (62.4)
Prior stroke, n (%)	2 (2.4)
Symptoms and functional class	
Reduced exercise tolerance, n (%)	81 (95.3)
Fatigue, n (%)	69 (81.2)
Leg swelling, n (%)	47 (55.3)
Pedal oedema, n (%)	30 (35.3)
Raised jugular venous pressure, n (%)	4 (4.7)
Crepitations, n (%)	8 (9.4)
NYHA class I, n (%)	6 (7.1)
NYHA class II, n (%)	39 (45.9)
NYHA class III, n (%)	30 (35.3)
NYHA class IV, n (%)	10 (11.8)
Haemodynamics	
Heart rate, beats/min, mean ± SD	85.0 ± 9.9
Systolic blood pressure, mmHg, mean ± SD	108.8 ± 22.8
Diastolic blood pressure, mmHg, mean ± SD	64.9 ± 6.6
Respiratory rate, breaths/min, mean ± SD	15.5 ± 1.2
Medications at enrolment	
RAS inhibitor (ACEi/ARB/ARNI), n (%)	68 (80.0)
Beta-blocker, n (%)	73 (85.9)
Mineralocorticoid receptor antagonist, n (%)	46 (54.1)
SGLT2 inhibitor, n (%)	38 (44.7)
Loop diuretic, n (%)	58 (68.2)
Statin, n (%)	76 (89.4)
Antiplatelet, n (%)	78 (91.8)
Laboratory findings	
Haemoglobin, g/dL, mean ± SD	12.7 ± 1.6
Anaemia (WHO criteria), n (%)	40 (47.1)
Serum creatinine, mg/dL, mean ± SD	1.6 ± 0.5
eGFR, mL/min/1.73 m², mean ± SD	52.4 ± 21.3
Fasting blood glucose, mmol/L, mean ± SD	6.6 ± 1.7
Two-hour postprandial glucose, mmol/L, mean ± SD	8.1 ± 1.6
HbA1c, %, mean ± SD	6.7 ± 1.6
NT-proBNP, pg/mL, median (IQR)	1840 (850–3420)
Echocardiographic characteristics	
LVEF, %, mean ± SD	32.1 ± 4.1
LV end-diastolic volume, mL, mean ± SD	162.4 ± 34.2
LV end-systolic volume, mL, mean ± SD	110.3 ± 28.6
LV end-diastolic volume index, mL/m², mean ± SD	91.8 ± 18.5
E/e′, mean ± SD	15.2 ± 4.1
Atrial fibrillation, n (%)	6 (7.1)
Mitral regurgitation severity*	
None or trivial, n (%)	28 (32.9)
Mild, n (%)	36 (42.4)
Moderate (functional/secondary), n (%)	21 (24.7)

Right ventricular functional parameters

Evidence of right ventricular dysfunction was frequent across multiple echocardiographic indices. Reduced TAPSE and S′ were each observed in 62.4% of patients, while reduced RV FAC was noted in 56.5%. Elevated MPI was present in 55.3%, prolonged IVRT in 57.6%, and reduced IVA in 31.8%. Impairment of RVGLS and RVFWS was detected in 76.5% and 71.8% of patients, respectively (Table 2). 

**Table 2:** Frequency of right ventricular dysfunction according to echocardiographic parameter Cut-off values for the conventional right ventricular systolic and diastolic indices were applied according to the echocardiographic recommendations current at the time of data collection. Thresholds for TAPSE (<17 mm), S′ (<10 cm/s), RV fractional area change (<35%), and myocardial performance index (≥0.55) follow the American Society of Echocardiography/European Association of Cardiovascular Imaging right-heart and chamber-quantification recommendations [7,14]; the TAPSE threshold derives specifically from the 2015 chamber-quantification update [14]. Isovolumic relaxation time (>30 ms) was assessed by tissue Doppler imaging and isovolumic acceleration (<1.1 m/s²), a relatively load-independent index of right ventricular contractility, was interpreted in line with established right-ventricular assessment literature [1,7]. Strain thresholds follow published reference values [16]. RV: right ventricular; TAPSE: tricuspid annular plane systolic excursion; S′: tricuspid annular peak systolic velocity.

Right ventricular parameter	Number of patients	Percentage
TAPSE (mm)		
Low (<17)	53	62.4
Normal (17–22)	32	37.6
Tricuspid annular peak systolic velocity, S′ (cm/s)		
Low (<10)	53	62.4
Normal (≥10)	32	37.6
RV fractional area change (%)		
Low (<35.0)	48	56.5
Normal (≥35.0)	37	43.5
Myocardial performance index		
High (≥0.55)	47	55.3
Normal (<0.55)	38	44.7
Isovolumic relaxation time (ms)		
Prolonged (>30)	49	57.6
Normal (≤30)	36	42.4
Isovolumic acceleration (m/s²)		
Low (<1.1)	27	31.8
Normal (≥1.1)	58	68.2
RV global longitudinal strain (%)		
Impaired (less negative than −17)	65	76.5
Normal (≤−17)	20	23.5
RV free wall strain (%)		
Impaired (less negative than −20)	61	71.8
Normal (≤−20)	24	28.2

Advanced echocardiographic findings

Ventricular interdependence, evidenced by interventricular septal flattening, was present in 15 patients (17.6%), intraventricular dyssynchrony in 9 (10.6%), and interventricular dyssynchrony in 21 (24.7%). An abnormal diastolic eccentricity index was observed in 18 patients (21.2%) and abnormalities in both diastole and systole in 8 (9.4%). Regional RV wall motion abnormality was identified in 19 patients (22.4%), most commonly involving the lateral wall (Table 3).

**Table 3:** Factor affecting right ventricular dysfunction of the study patients Ventricular interdependence was assessed from the pattern of interventricular septal flattening. Intraventricular dyssynchrony was defined by a septal-to-posterior wall motion delay ≥130 ms and interventricular dyssynchrony by an interventricular mechanical delay >40 ms [17]. Left ventricular geometry was characterised using the eccentricity index in diastole and at end-systole [14]. Regional right ventricular wall motion abnormality was assessed qualitatively in standard apical and subcostal views [7,14]. Atrial fibrillation is reported in Table 1. IVMD: interventricular mechanical delay; LV: left ventricular; RV: right ventricular; SD: standard deviation; SPWMD: septal-to-posterior wall motion delay.

Parameter	Number of patients	Percentage
Ventricular interdependence (septal flattening)		
Present (any)	15	17.6
Systole only	2	2.4
Diastole only	9	10.6
Both systole and diastole	4	4.7
Absent	70	82.4
Ventricular dyssynchrony		
Intraventricular dyssynchrony (SPWMD ≥130 ms)	9	10.6
Interventricular dyssynchrony (IVMD >40 ms)	21	24.7
Left ventricular geometry (eccentricity index)		
Abnormal in diastole	18	21.2
Abnormal in both diastole and systole	8	9.4
Normal	59	69.4
RV regional wall motion abnormality		
Lateral wall	11	12.9
Inferior wall	5	5.9
Anterior wall	3	3.5
Absent	66	77.6

Pulmonary pressures and their association with right ventricular indices

Elevated PASP (>35 mmHg) was observed in 33 patients (38.8%), while 52 (61.2%) had values within the normal range; pressures of 35-50 mmHg were recorded in 27.1%, 51-70 mmHg in 9.4%, and above 70 mmHg in 2.4% (Table 4). On logistic regression, elevated PASP was associated with abnormal S′ (OR 6.39; 95% CI 1.11-36.73; p = 0.038) and with prolonged IVRT (OR 8.79; 95% CI 1.72-44.93; p = 0.009). No significant association was demonstrated with abnormal TAPSE, FAC, MPI, IVA, RVGLS, or RVFWS (Table 5).

**Table 4:** Distribution of the study patients according to pulmonary artery systolic pressure (n = 85) Pulmonary artery systolic pressure (PASP) was estimated non-invasively from the peak tricuspid regurgitation jet velocity using the modified Bernoulli equation, with the addition of right atrial pressure estimated from the diameter and respiratory collapsibility of the inferior vena cava [7]. Values were categorised into predefined bands, with PASP >35 mmHg considered elevated. Under current guidance, an elevated Doppler-derived pressure confers a probability of pulmonary hypertension rather than establishing the diagnosis [8].

Pulmonary artery systolic pressure (mmHg)	Number of patients	Percentage
Normal (<35)	52	61.2
35–50	23	27.1
51–70	8	9.4
>70	2	2.4

**Table 5:** Logistic regression analysis of right ventricular functional indices and elevated pulmonary artery systolic pressure Binary logistic regression was performed with each right ventricular functional index modelled separately as the dependent variable and elevated pulmonary artery systolic pressure (>35 mmHg) as the predictor. Models contained a single predictor and are therefore unadjusted. The significance of the predictor was assessed using the Wald test; the Wald χ² statistic (df = 1) is reported alongside the odds ratio (OR), 95% confidence interval (CI), and two-sided p-value. A p-value <0.05 was considered statistically significant. CI: confidence interval; OR: odds ratio; RV: right ventricular; S′: tricuspid annular peak systolic velocity; TAPSE: tricuspid annular plane systolic excursion.

Right ventricular index	Wald χ²	OR	95% CI	p value
Abnormal TAPSE	0.74	2.06	0.40–10.68	0.390
Abnormal S′	4.30	6.39	1.11–36.73	0.038
Abnormal RV fractional area change	0.20	1.38	0.34–5.61	0.651
High myocardial performance index	0.31	1.48	0.37–5.95	0.581
Prolonged isovolumic relaxation time	6.78	8.79	1.72–44.93	0.009
Abnormal isovolumic acceleration	0.41	0.59	0.11–3.02	0.522
Abnormal RV global longitudinal strain	0.07	1.28	0.20–8.14	0.793
Abnormal RV free wall strain	0.72	2.08	0.39–11.18	0.394

Regional right ventricular wall motion abnormality

Regional RV wall motion abnormality was not significantly associated with any of the right ventricular functional indices for which valid estimates could be obtained (Table 6). Models for TAPSE and S′ failed to converge because of quasi-complete separation and are not reported.

**Table 6:** Logistic regression analysis of right ventricular functional indices and regional right ventricular wall motion abnormality Binary logistic regression was used to assess the association between right ventricular regional wall motion abnormality and each abnormal right ventricular functional index, with each index modelled separately as the dependent variable. Models contained a single predictor and are therefore unadjusted. The Wald χ² statistic (df = 1) is reported alongside the odds ratio (OR), 95% confidence interval (CI), and two-sided p-value. Models for TAPSE and S′ are not shown; estimation failed owing to quasi-complete separation arising from zero or near-zero cell counts. CI: confidence interval; OR: odds ratio; RV: right ventricular; S′: tricuspid annular peak systolic velocity; TAPSE: tricuspid annular plane systolic excursion.

Right ventricular index	Wald χ²	OR	95% CI	p value
Abnormal RV fractional area change	0.02	1.13	0.21–6.10	0.887
High myocardial performance index	0.09	1.27	0.26–6.31	0.770
Prolonged isovolumic relaxation time	0.80	2.39	0.36–15.98	0.368
Abnormal isovolumic acceleration	0.06	0.82	0.16–4.23	0.812

## Discussion

RV dysfunction is increasingly recognised as an important correlate of prognosis in left-sided heart disease, including ischaemic cardiomyopathy and HFrEF [1,2,5,6]. In this study, RV dysfunction was highly prevalent among patients with old MI and reduced LVEF. Between 56% and 62% of patients exhibited abnormalities in conventional RV indices (TAPSE, S′, and FAC). Strain-based assessment demonstrated an even higher burden of RV impairment, with abnormal RVGLS in more than three-quarters and abnormal RVFWS in almost as many participants. These findings indicate substantial RV involvement in ischaemic cardiomyopathy and support systematic RV evaluation in this population.

Although conventional RV parameters are widely used, their sensitivity for early or subclinical RV dysfunction can be limited, particularly given the complex geometry of the right ventricle [7,8]. In contrast, speckle-tracking-derived RV strain provides a quantitative assessment of longitudinal myocardial deformation, defined and standardised by consensus documents [9,10]. The high prevalence of abnormal RV strain in this cohort aligns with prior evidence that RV strain can detect dysfunction even when conventional indices appear relatively preserved [11,16]. RV strain parameters have also been associated with adverse clinical outcomes in ischaemic settings [12,18].

Elevated PASP was the variable most consistently associated with abnormal RV indices in this cohort. This association is biologically plausible: pulmonary hypertension due to left heart disease increases RV afterload and contributes to progressive RV systolic and diastolic impairment [4]. Under the current framework, however, an elevated Doppler-derived pressure confers a probability of pulmonary hypertension rather than establishing the diagnosis [8], and our findings are therefore presented as an association between elevated estimated pulmonary pressure and abnormal RV indices rather than as evidence of pulmonary hypertension per se.

Reconciliation with current guideline thresholds

Echocardiographic data for this cohort were acquired in 2021-2022 using cut-offs current at that time and were subsequently re-examined against the 2025 ASE right-heart framework. The TAPSE threshold (<17 mm) and the FAC threshold (<35%) are unchanged, although both are now reported as graded severity categories rather than binary states. The S′ threshold has been refined to <9.5 cm/s, marginally lower than the <10 cm/s applied here, so the present estimate of abnormal S′ (62.4%) may modestly overstate the prevalence that the current threshold would yield. The PASP bands used in this study (<35, 35-50, 51-70, >70 mmHg) correspond closely to the 2025 severity ranges for right ventricular systolic pressure, supporting comparability. The 2025 document also places less weight on composite Doppler indices such as MPI and IVA than on TAPSE, FAC, S′, strain, and three-dimensional RV ejection fraction, which should be borne in mind when interpreting those parameters in this cohort [8].

Clinical implications

The high burden of right ventricular involvement observed here has several practical consequences for the management of ischaemic cardiomyopathy. RV dysfunction is an established independent predictor of mortality, hospitalisation, and reduced functional capacity in chronic heart failure [2,5,6], so its identification refines risk stratification beyond LVEF alone in a group already recognised as high risk. Recognition also has direct therapeutic relevance. Patients with impaired RV function tolerate volume shifts poorly and are more susceptible to diuretic-induced preload reduction and hypotension, which may limit the uptitration of renin-angiotensin system inhibitors and beta-blockers; in our cohort, mineralocorticoid receptor antagonists and sodium-glucose cotransporter-2 inhibitors were prescribed to only 54.1% and 44.7% of patients, respectively, and RV assessment may help identify those in whom cautious, individualised titration rather than omission is appropriate. RV function additionally informs candidacy for and response to device therapy and advanced heart failure interventions, and pre-existing RV impairment is relevant when considering cardiac resynchronisation therapy or, in selected patients, mechanical circulatory support [2]. Finally, because elevated PASP was the only variable showing a nominal association with abnormal RV indices in this cohort, systematic attention to pulmonary haemodynamics, congestion control, rate and rhythm management, and treatment of contributory functional mitral regurgitation represents a modifiable pathway through which RV loading may be influenced.

Potential role of strain imaging

Deformation imaging offers a quantitative, angle-independent measure of RV longitudinal function that is less dependent on the geometric assumptions limiting conventional indices, and the 2025 ASE guideline accordingly assigns RV free wall strain a defined role with graded severity categories [8]. In external cohorts, RV strain has been associated with adverse outcomes in ischaemic and non-ischaemic populations and has identified impairment in patients whose conventional indices appeared preserved [11,12,18]. In the present study, abnormalities of global longitudinal and free wall strain were the most frequently observed of all indices examined; however, no comparative discrimination or reclassification analysis was performed, and these frequencies alone cannot establish that strain outperforms conventional measures in this setting. The clinically relevant implication is therefore a practical one: incorporating RV strain into the routine echocardiographic protocol for patients with prior infarction and reduced ejection fraction is feasible on contemporary platforms, adds little acquisition time, and generates a quantitative value suitable for serial follow-up during therapy uptitration. Whether strain-guided assessment alters management decisions or improves outcomes in this population requires prospective study with clinical endpoints and formal comparison against conventional indices.

Regional wall motion and structural findings

Visually assessed regional RV wall motion abnormality did not show a significant association with any RV functional index. Ventricular interdependence, mechanical dyssynchrony, and altered LV geometry were documented descriptively but were not modelled, and no inference about their independent contribution can be drawn from these data. The limited concordance between subjective RV regional wall motion assessment and quantitative RV indices is consistent with established mechanistic accounts of RV loading in chronic left heart disease [3,4,19] and reinforces the value of objective quantitative measures [7-10].

Limitations

This study was conducted at a single centre with a relatively small sample size, which may limit generalisability. The cross-sectional design precludes causal inference, and although enrolment was consecutive, referral to a tertiary cardiac centre may introduce selection bias towards more symptomatic patients.

Several limitations relate to echocardiographic assessment. Formal intra- and inter-observer reproducibility analysis for the right ventricular indices was not performed; measurement variability may therefore have influenced the reported prevalence estimates, particularly for the speckle-tracking-derived strain parameters, which are more sensitive to image quality and endocardial border tracking than conventional measures. Strain measurements were obtained on a single-vendor platform, and despite progress in inter-vendor standardisation, absolute strain values are not directly interchangeable between systems; the reference thresholds applied here were derived largely from cohorts studied on other platforms, which may affect the classification of individual patients as normal or impaired. Because reduced ejection fraction and prior infarction were eligibility criteria assessed echocardiographically, the operator could not be blinded to these features, and measurement of right ventricular indices may have been subject to expectation bias. Five patients were excluded after enrolment because of inadequate image quality for speckle-tracking analysis, which may have selected against those with the poorest acoustic windows. Left ventricular strain analysis and three-dimensional echocardiographic assessment of right ventricular volumes and ejection fraction were not performed, and right ventricular strain was analysed dichotomously rather than using the graded severity categories introduced in the 2025 American Society of Echocardiography guideline.

Pulmonary artery systolic pressure was estimated by Doppler echocardiography rather than by invasive haemodynamic measurement. Peak tricuspid regurgitation velocity was not reported separately, precluding application of the current probability-based approach to pulmonary hypertension. Cut-off values applied were those current at the time of data collection, and the subsequent refinement of the tricuspid annular systolic velocity threshold is discussed above. Moderate functional mitral regurgitation was present in approximately one-quarter of the cohort and may have contributed to elevated pulmonary pressures; its contribution cannot be separated from that of left ventricular dysfunction in a cross-sectional design.

Coronary angiography was not mandated by the protocol and had been performed in 68.2% of patients, so the extent and distribution of coronary disease were not uniformly characterised, and 45.9% of the cohort had not undergone revascularisation, which may have contributed to the observed burden of right ventricular dysfunction.

Several statistical limitations warrant emphasis. Regression models contained a single predictor and were unadjusted, so residual confounding cannot be excluded. With 85 participants and a modest number of events per variable, estimates are susceptible to sparse-data bias, and two models could not be estimated because of quasi-complete separation arising from zero or near-zero cell counts. Twelve separate regression models were fitted across the two analyses without correction for multiplicity; neither of the two nominally significant associations would remain significant after Bonferroni correction, and these results should therefore be regarded as hypothesis-generating rather than confirmatory. No comparative discrimination or reclassification analysis was undertaken, so the relative diagnostic performance of strain-based and conventional indices cannot be inferred from these data.

Most importantly, clinical outcomes were not captured. The cross-sectional design included no follow-up for heart failure hospitalisation, cardiovascular death, or all-cause mortality, and the present findings therefore describe the burden and echocardiographic correlates of right ventricular dysfunction rather than its prognostic consequence in this population. Prospective longitudinal follow-up of this cohort, with adjudicated clinical endpoints, would allow the right ventricular indices reported here to be tested against long-term outcomes.

## Conclusions

Right ventricular dysfunction is highly prevalent among patients with old myocardial infarction and reduced left ventricular ejection fraction. Abnormalities were frequently detected across both conventional and strain-based echocardiographic parameters, with right ventricular global longitudinal strain showing the highest frequency of impairment. Elevated pulmonary artery systolic pressure showed a nominal association with abnormal right ventricular indices, consistent with a contributory role for pulmonary haemodynamics in right ventricular involvement following myocardial infarction, although this finding did not survive correction for multiple testing and requires confirmation. These findings support comprehensive right ventricular assessment, including strain analysis, in this high-risk population, while prospective studies with clinical endpoints and formal comparative analysis are required to establish whether such assessment improves risk stratification or outcomes.

## References

[1] Haddad F, Hunt SA, Rosenthal DN, Murphy DJ (2008). Right ventricular function in cardiovascular disease, part I: anatomy, physiology, aging, and functional assessment of the right ventricle. Circulation.

[2] Konstam MA, Kiernan MS, Bernstein D (2018). Evaluation and management of right-sided heart failure: a scientific statement from the American Heart Association. Circulation.

[3] Haddad F, Doyle R, Murphy DJ, Hunt SA (2008). Right ventricular function in cardiovascular disease, part II: pathophysiology, clinical importance, and management of right ventricular failure. Circulation.

[4] Guazzi M, Borlaug BA (2012). Pulmonary hypertension due to left heart disease. Circulation.

[5] Ghio S, Gavazzi A, Campana C (2001). Independent and additive prognostic value of right ventricular systolic function and pulmonary artery pressure in patients with chronic heart failure. J Am Coll Cardiol.

[6] Polak JF, Holman BL, Wynne J, Colucci WS (1983). Right ventricular ejection fraction: an indicator of increased mortality in patients with congestive heart failure associated with coronary artery disease. J Am Coll Cardiol.

[7] Rudski LG, Lai WW, Afilalo J (2010). Guidelines for the echocardiographic assessment of the right heart in adults: a report from the American Society of Echocardiography endorsed by the European Association of Echocardiography, a registered branch of the European Society of Cardiology, and the Canadian Society of Echocardiography. J Am Soc Echocardiogr.

[8] Mukherjee M, Rudski LG, Addetia K (2025). Guidelines for the echocardiographic assessment of the right heart in adults and special considerations in pulmonary hypertension: recommendations from the American Society of Echocardiography. J Am Soc Echocardiogr.

[9] Badano LP, Kolias TJ, Muraru D (2018). Standardization of left atrial, right ventricular, and right atrial deformation imaging using two-dimensional speckle tracking echocardiography: a consensus document of the EACVI/ASE/Industry Task Force to standardize deformation imaging. Eur Heart J Cardiovasc Imaging.

[10] Voigt JU, Pedrizzetti G, Lysyansky P (2015). Definitions for a common standard for 2D speckle tracking echocardiography: consensus document of the EACVI/ASE/Industry Task Force to standardize deformation imaging. Eur Heart J Cardiovasc Imaging.

[11] Tadic M, Nita N, Schneider L (2021). The predictive value of right ventricular longitudinal strain in pulmonary hypertension, heart failure, and valvular diseases. Front Cardiovasc Med.

[12] Prihadi EA, van der Bijl P, Dietz M (2019). Prognostic implications of right ventricular free wall longitudinal strain in patients with significant functional tricuspid regurgitation. Circ Cardiovasc Imaging.

[13] Thygesen K, Alpert JS, Jaffe AS, Chaitman BR, Bax JJ, Morrow DA, White HD (2018). Fourth universal definition of myocardial infarction (2018). Circulation.

[14] Lang RM, Badano LP, Mor-Avi V (2015). Recommendations for cardiac chamber quantification by echocardiography in adults: an update from the American Society of Echocardiography and the European Association of Cardiovascular Imaging. J Am Soc Echocardiogr.

[15] Nagueh SF, Smiseth OA, Appleton CP (2016). Recommendations for the evaluation of left ventricular diastolic function by echocardiography: an update from the American Society of Echocardiography and the European Association of Cardiovascular Imaging. J Am Soc Echocardiogr.

[16] Fine NM, Chen L, Bastiansen PM, Frantz RP, Pellikka PA, Oh JK, Kane GC (2015). Reference values for right ventricular strain in patients without cardiopulmonary disease: a prospective evaluation and meta-analysis. Echocardiography.

[17] Gorcsan J 3rd, Abraham T, Agler DA (2008). Echocardiography for cardiac resynchronization therapy: recommendations for performance and reporting - a report from the American Society of Echocardiography Dyssynchrony Writing Group endorsed by the Heart Rhythm Society. J Am Soc Echocardiogr.

[18] Park SJ, Park JH, Lee HS (2015). Impaired RV global longitudinal strain is associated with poor long-term clinical outcomes in patients with acute inferior STEMI. JACC Cardiovasc Imaging.

[19] Lahm T, Douglas IS, Archer SL (2018). Assessment of right ventricular function in the research setting: knowledge gaps and pathways forward. An official American Thoracic Society research statement. Am J Respir Crit Care Med.

